# Neutrophil α-defensin-1 is present in human stroke thrombi and induces NETosis *in vitro*

**DOI:** 10.3389/fimmu.2026.1821379

**Published:** 2026-05-29

**Authors:** Edward Theodore Littleton, Samuel George Thomas, Eleanor Woodhead, Julie Rayes, Alexander Brill

**Affiliations:** 1 Department of Cardiovascular Sciences, College of Medicine and Health, School of Medical Sciences, University of Birmingham, Birmingham, United Kingdom; 2 Department of Neurology, University Hospitals Birmingham NHS Foundation Trust, Queen Elizabeth Hospital Birmingham, Birmingham, United Kingdom

**Keywords:** immunothrombosis, ischemic stroke, neutrophil extracellular traps (NETs), thrombus composition, α-defensin-1

## Abstract

**Background:**

Ischemic stroke thrombi contain complex immunothrombotic components, including neutrophil extracellular traps (NETs), which contribute to thrombus stability and resistance to fibrinolysis. However, the endogenous triggers of NETosis within sterile stroke thrombi remain poorly understood. Neutrophil α-defensins are cationic peptides with emerging pro-thrombotic roles, but their presence and function in human stroke clots have not been fully characterized. This study investigated whether α-defensin-1 is present in ischemic stroke thrombi and whether it can induce NETosis.

**Methods:**

Thrombi were collected from 6 patients undergoing mechanical thrombectomy for acute ischemic stroke and analyzed using immunofluorescence to assess α-defensin-1 localization and NET markers. Human neutrophils isolated from healthy donors were stimulated *in vitro* with α-defensin-1, phorbol 12-myristate 13-acetate, or vehicle control. NETosis was quantified as the percentage of citrullinated histone H3-positive nuclei using standardized threshold-based ImageJ analysis.

**Results:**

α-Defensin-1 was detected in all analyzed thrombi, predominantly in extracellular regions enriched with neutrophils and less abundant in erythrocyte-rich areas. NETs were a prominent feature of thrombi, as demonstrated by citrullinated histone H3 staining. *In vitro*, exposure to recombinant α-defensin-1 significantly increased NETosis at 10 µg/mL compared with unstimulated controls, whereas lower concentrations showed no significant effect. These findings indicate that α-defensin-1 is a structural component of stroke thrombi and induces NETosis *in vitro*.

**Conclusion:**

α-Defensin-1 may be an underappreciated component of stroke thrombi that induces NETosis *in vitro*, suggesting a link between neutrophil degranulation and thrombus composition.

## Introduction

Ischemic stroke, resulting from the thromboembolic occlusion of cerebral arteries, remains among the leading causes of mortality and long-term disability worldwide (1). A central pathological feature of acute ischemic stroke is the formation of intravascular thrombi that obstruct blood flow and injure downstream brain tissue. Recent histopathological studies of retrieved stroke thrombi have revealed a heterogeneous composition of fibrin, platelets, red blood cells (RBCs), von Willebrand factor, and leukocyte-derived components such as neutrophil extracellular traps (NETs) (2). This growing understanding of thrombus composition has underscored the concept of immunothrombosis, wherein the interplay between the coagulation system and immune cells contributes to thrombus formation and persistence. In particular, neutrophils and NETs have emerged as key contributors to clot stability and resistance to thrombolytic therapies (3).

NETs are web-like extracellular networks of decondensed chromatin decorated with histones and neutrophil granule proteins (4). The process of NET formation (NETosis) is implicated in a broad range of thromboinflammatory conditions, including sepsis, myocardial infarction, and ischemic stroke (5–7). NETs not only trap pathogens but also provide a scaffold for platelets and fibrin, thereby promoting thrombus growth and stability. Consistently, NETs have been detected in virtually all analyzed human stroke thrombi (often concentrated at the thrombus periphery), and digesting NET DNA with DNase has been shown to facilitate tissue plasminogen activator (tPA)-mediated clot lysis (3, 4). These findings suggest that NETs substantially reinforce the thrombus extracellular matrix, contributing to thrombus resistance to both mechanical retrieval and fibrinolysis (8). However, the upstream triggers of NETosis within stroke clots remain incompletely understood, as classical NETosis stimuli (microbes, damage-associated molecules, or activated platelets) alone do not fully explain the robust NET formation observed in sterile, occlusive human thrombi. This gap in knowledge points to additional endogenous factors in the thrombus microenvironment that drive NETosis.

Alpha-defensins are small cationic peptides predominantly stored in neutrophil azurophilic granules, best known for their potent antimicrobial activity (9). Beyond direct host defense, defensins are increasingly recognized as immunomodulatory mediators that regulate inflammation and immunity (10). Notably, accumulating evidence links neutrophil α-defensins (also called human neutrophil peptides, HNP1-3) to thrombo-inflammatory processes. Activated neutrophils release α-defensins during inflammation, raising plasma α-defensin concentrations markedly (from sub-nanomolar basal levels to tens of micromolar in acute infection). Elevated α-defensin levels have been associated with cardiovascular diseases – correlations with myocardial infarction, ischemic stroke, and atherosclerotic disease severity have been reported (11, 12). Mechanistic studies demonstrate that α-defensins can directly promote thrombosis: they bind to blood vessels and nascent clots, accelerate fibrin polymerization, increase fibrin fiber density, and inhibit fibrinolysis (13). *In vivo*, mice engineered to express human α-defensin develop larger, more occlusive thrombi that are resistant to anticoagulation and fibrinolysis (13). These observations suggest that α-defensins possess pro-thrombotic properties that extend beyond their antimicrobial role. Nevertheless, the presence and function of α-defensins within human ischemic stroke thrombi have not been previously explored, and it is unknown whether these neutrophil-derived peptides influence NETosis in the context of stroke.

Here, we report observations that support a potential association between the presence of α-defensin-1 in human ischemic stroke thrombi and its ability to induce NET-associated changes in neutrophils *in vitro*. First, we demonstrate that α-defensin-1 is abundantly present in human ischemic stroke thrombi, co-localizing with neutrophils in the clot. Second, we show that α-defensin-1 induces NETosis in isolated human neutrophils. To our knowledge, this is the first evidence that α-defensins are integral components of stroke thrombi and can potentially act as endogenous triggers of NET formation. These findings support a potential mechanistic link between neutrophil granule release and NET-driven thrombus stabilization in stroke.

## Methods

### Patient recruitment and thrombus collection

Adult patients presenting to Queen Elizabeth Hospital Birmingham with acute ischemic stroke due to occlusion of the middle cerebral artery or basilar artery confirmed on CT angiography (from the aortic arch to the cranial vertex), were considered eligible for mechanical thrombectomy if treatment could commence within 6 hours of stroke onset. Patients with visible infarction on non-contrast CT were excluded. Where the time of stroke onset exceeded 6 hours or was unclear, patients were eligible if CT perfusion imaging demonstrated a substantial penumbra with a small infarct core.

Mechanical thrombectomy was performed using standard aspiration or stent-retriever techniques. Thrombi were collected intra-procedurally and processed within 30 minutes. Retrospective consent for the use of thrombus in subsequent laboratory experiments was obtained from patients or their consultees in accordance with the ethically approved protocol for this study (NHS Health Research Authority, REC reference 23/LO/0249, IRAS project ID: 317904). Clinical and radiological data were retrieved from hospital records.

### Immunohistochemistry of stroke thrombi

Freshly retrieved thrombi were immersed in optimal cutting temperature (OCT) compound (VWR International, Radnor, USA), snap-frozen in liquid nitrogen, and stored at -80 °C. Six-micron cryosections were mounted on glass slides, fixed in 4% formaldehyde (CellPath, Powys, UK) for 10 min, and blocked with 1% bovine serum albumin (BSA) (First Link UK Ltd, Wolverhampton, UK) in phosphate buffered saline (PBS) (Sigma-Aldrich, Saint Louis, USA) for 1 h.

Slides were incubated with rabbit polyclonal antibodies against α-defensin-1 (NBP3-05562, Bio-techne Ltd, Abingdon, UK) or citrullinated histone H3 (CitH3) (ab5103, Abcam, Cambridge, UK), both at 1:100 dilution in PBS with 1% BSA, followed by fluorescently-conjugated goat anti-rabbit secondary antibodies (Alexa 488 or 647, 1:1000; Thermo Fisher Scientific Inc, Waltham, USA). Slides were also stained with Alexa 488-conjugated mouse monoclonal antibodies against neutrophil elastase (BD Pharmingen, San Diego, USA) and Alexa 568-conjugated mouse monoclonal anti-CD235 antibody (Thermo Fisher Scientific Inc, Waltham, USA), both at 1:100 dilution, followed by nuclear staining with Hoechst 33342 (1:10000; Fisher Scientific, Loughborough, UK). Imaging was performed using an epifluorescent microscope (Definite Focus 2, Colibri 7 LED illumination source, Hamamatsu Flash 4 V2 sCMOS camera, Zen 2.3 Pro software). Fluorescence signal intensity of α-defensin-1 was measured using ImageJ (14) to compare leukocyte-rich and erythrocyte-rich regions of thrombi. For quantification of NETs, the number of fluorescently stained cells in each image was determined with fixed threshold settings and a particle size filter. The proportion of CitH3-positive cells relative to the total number of Hoechst-positive nuclei was calculated for each image.

### Neutrophil isolation

All experiments with blood from healthy volunteers were approved by the institutional ethical review board (ERN_11-0175AP26) and performed in accordance with the principles of the Declaration of Helsinki. Peripheral blood (20–30 mL) was collected from cubital vein into EDTA tubes (Vacuette, Greiner Bio-One GmbH, Kremsmünster, Austria). Neutrophils were isolated via density gradient centrifugation using a layered Histopaque-1119/1077 system (Sigma-Aldrich, Saint Louis, USA). After centrifugation at 1200 g for 45 min, the interface layer was collected, diluted in pre-warmed RPMI 1640 medium (Sigma-Aldrich, Saint Louis, USA), and centrifuged at 400 g for 8 min.

The pellet was treated with Ammonium-Chloride-Potassium (ACK) lysing buffer (Gibco, Fisher Scientific, Loughborough, UK) 1 mL, for 3 min, on ice, washed with RPMI 1640, centrifuged at 400 g for 5 min, and resuspended in fresh medium. Concentration of the cell suspension was determined using a Thermo Fisher Countess 3 automated cell counter, and cell concentration was adjusted to approximately 5x10^5^ cells/mL.

### *In vitro* NETosis assay

All experiments were started within 2 h of blood collection. Neutrophils (150,000 cells in 300 µL) were seeded onto 13mm glass coverslips coated in poly-L-lysine 0.1% (Sigma-Aldrich, Saint Louis, USA). After 30 minutes incubation at 37 °C, cells were exposed to α-defensin-1, at either 1 or 10 µg/mL (PeproTech, Thermo Fisher Scientific Inc, Waltham, USA), phorbol 12-myristate 13-acetate (PMA) 100 nM (Sigma-Aldrich, Saint Louis, USA), or vehicle, and were incubated for 90 min at 37 °C. Then, the media were removed, cells were fixed in 4% formaldehyde for 15 minutes and blocked in 10% fetal bovine serum (FBS) (Sigma-Aldrich, Saint Louis, USA) in PBS. Slides were incubated with rabbit anti-CitH3 (1:100 in 10% FBS), followed by fluorescently labeled (Alexa 647) goat anti-rabbit (1:1000) antibody. Nuclei and DNA were counterstained with Hoechst 33342 at 1:10000 dilution. Coverslips were mounted on glass slides for imaging.

### Image acquisition and quantification for *in vitro* NETosis assay

Images were acquired using Zeiss Axio Observer 7 inverted epifluorescence microscope at x20 magnification. Eight images were taken per coverslip: four near the edge and four from the central region. The number of fluorescent-stained cells in each image was quantified using ImageJ software (14), with fixed threshold settings and particle size filter. The number of CitH3-positive cells relative to the total number of Hoechst-positive nuclei was calculated as a percentage per image.

### Statistics

**Figure 1:**
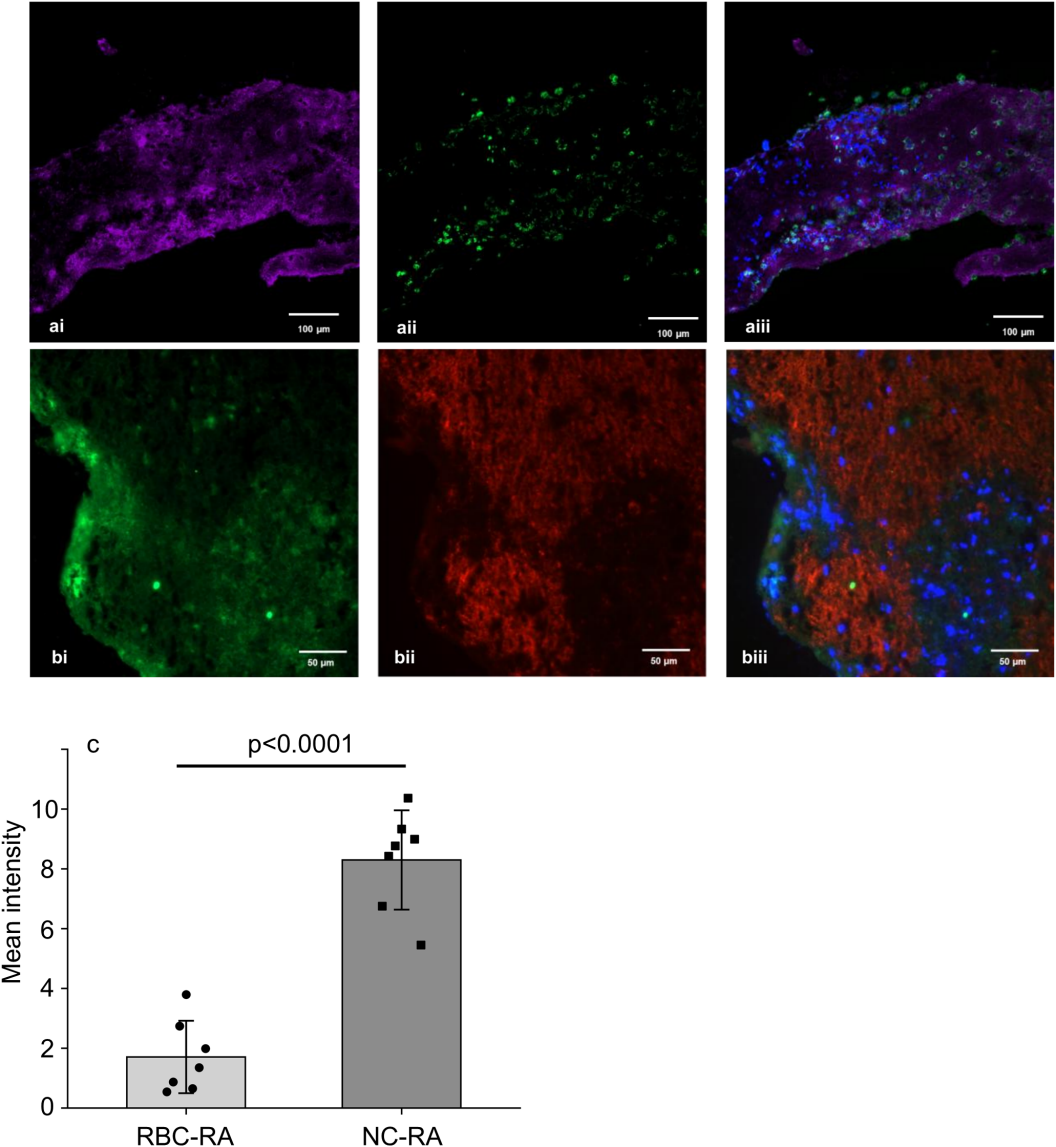
Distribution of α-defensin-1 in human ischemic stroke thrombi. **(A)** Representative immunofluorescence images of thrombus sections showing the partial co-localization of α-defensin-1 (magenta) and neutrophil elastase (green). α-Defensin-1 demonstrates a predominantly diffuse extracellular staining pattern (ai), whereas neutrophil elastase exhibits a punctate intracellular distribution (aii). Merged images with nuclear counterstain Hoechst 33342 (blue) are shown in (aiii); x20 magnification, scale bar 100 µm; n = 6. **(B)** Representative images illustrating the spatial relationship between α-defensin-1 (green) and erythrocyte-rich (red) regions. α-defensin-1 staining (bi) is primarily observed in thrombus areas with fewer CD235^+^ red blood cells (bii) and increased numbers of Hoechst 33342^+^ (blue) nucleated cells (biii), indicating enrichment within leukocyte-rich regions of the clot. x40 magnification, scale bar 50 µm; n = 6. **(C)** Mean intensities of α-defensin-1 signal in areas rich in nucleated cells (NC-RA) vs. red blood cell-rich areas (RBC-RA).

**Figure 2:**
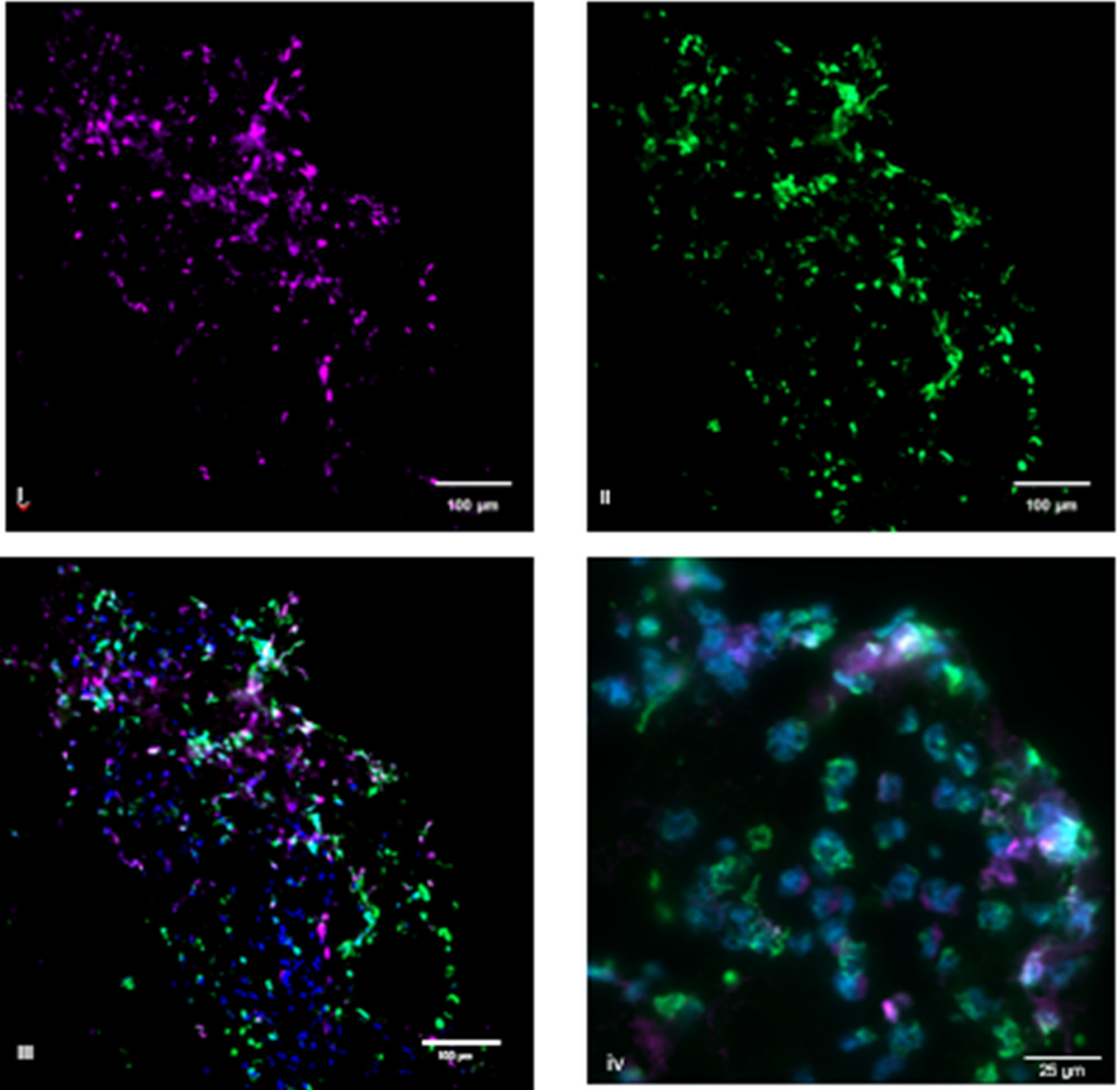
NETosis in human ischemic stroke thrombi. Representative immunofluorescence images of thrombus sections demonstrating markers of neutrophil extracellular trap (NET) formation. CitH3^+^ cells (i) comprise a subset of neutrophil elastase-positive cells (ii). Neutrophil elastase^+^ cells represent a major proportion of the nucleated cells (iii). (iv) High-magnification image illustrates CitH3-positive decondensed chromatin with extracellular extension, consistent with NET morphology; n = 6 thrombi from different donors.

For all analyses, the donor was used as the unit of biological replication. Eight fields of view per condition were averaged to generate a single value for each individual blood donor, thereby avoiding pseudoreplication. For thrombus image analysis (**Figure 1**), differences between leukocyte-rich and erythrocyte-rich regions were assessed using a two-tailed Student’s t-test. For *in vitro* experiments (**Figure 2**), nonparametric analyses were performed due to small sample sizes. Differences between groups were assessed using the Kruskal-Wallis test, followed by pairwise Mann-Whitney U tests.

Data are presented as mean ± SEM unless otherwise stated; **Figure 2** shows individual median values. All tests were two-tailed, and p < 0.05 was considered statistically significant. Analyses were performed using GraphPad Prism software.

## Results

### Patient characteristics

Thrombi were obtained from six patients with acute ischemic stroke undergoing mechanical thrombectomy. All occlusions were located in the M1 segment of the middle cerebral artery. The median age was 61 years (range 48-83), with five female and one male patient. The median NIHSS score on presentation was 14 (range 6-25), and the median time to clot retrieval was 6 hours (range 4-8). Two patients received intravenous thrombolysis. Three of the four non-lysed patients were on Factor Xa inhibitors, while one was excluded from thrombolysis due to delayed presentation. Relevant comorbidities included treated lymphoma, prior pulmonary embolism, recent myocarditis with ventricular thrombus, hypertension, and epilepsy.

### α-defensin-1 is abundantly present in human stroke thrombi

Immunofluorescent staining revealed α-defensin-1 positivity in all thrombi analyzed. The staining pattern was diffuse extracellular signal extending beyond neutrophil boundaries, in contrast to the more punctate, intracellular pattern observed for neutrophil elastase (**Figure 1A**). α-Defensin-1 was concentrated in the outer regions of thrombi, where nucleated cells were more abundant, and was notably less present in erythrocyte-rich, CD235-positive, core areas (**Figure 1B**). Quantitative image analysis confirmed significantly higher α-defensin-1 signal intensity in leukocyte-rich regions compared with erythrocyte-dense areas (**Figure 1C**). Regions enriched in nucleated cells, which exhibited higher α-defensin-1 signal intensity, also contained abundant CitH3-positive cells, indicating spatial co-enrichment of α-defensin-1 and NET-associated features within thrombi.

### NETs are a prominent component of stroke thrombi

Cells positive for CitH3, a well-established marker of NETosis, were detected in all thrombus specimens and comprised 26.7 ± 6.3% (mean ± SEM) of nucleated cells in the thrombi. These cells largely overlapped with neutrophil elastase-positive cells, suggesting that CitH3+ cells likely represented a significant subset of neutrophils present (**Figure 2** i–iii). High-magnification images demonstrated CitH3-positive cells with decondensed and dispersed chromatin, consistent with NET structures, in association with neutrophil staining (**Figure 2** iv). Because of antibody species limitations, direct co-localization of CitH3 and α-defensin-1 could not be assessed on the same slide.

### α-defensin-1 induces NETosis *in vitro*

**Figure 3:**
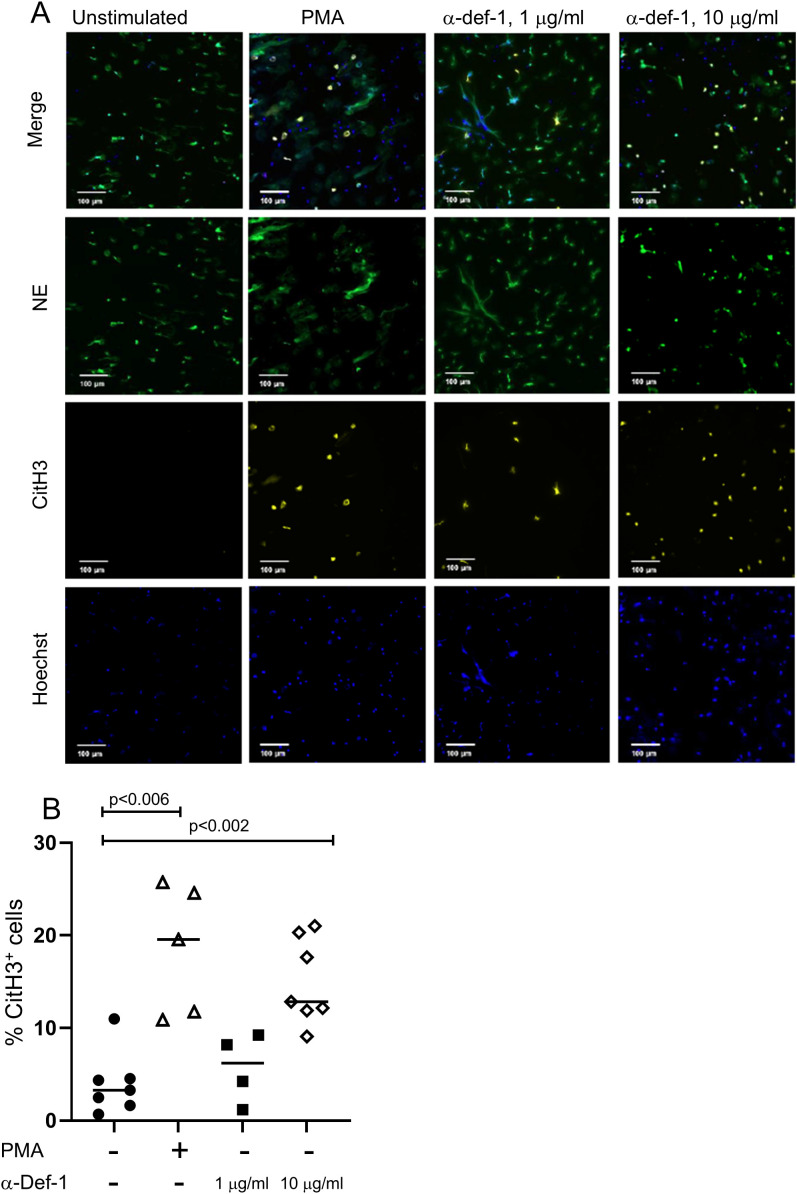
*In vitro* induction of NETosis by α-defensin-1. **(A)** Representative immunofluorescence images of isolated human neutrophils following 90-minute stimulation with PMA (100 nM) or α-defensin-1 (1 or 10 µg/mL). Neutrophil elastase (NE, green), CitH3 (amber) and DNA (Hoechst, blue) staining is presented. x20 magnification, scale bar 100 µm. Images are representative of 4 independent donors. **(B)** Quantification of NETosis across stimulation conditions. The number of CitH3^+^ cells was expressed as a percentage of total Hoechst 33342^+^ nuclei per field of view. Individual data points are shown together with mean values and SEM. Each dot represents one biological replicate (individual donor), derived from the median of multiple fields of view; n = 4–8.

NETosis was assessed by quantifying citrullinated histone H3 (CitH3) positivity, supported by neutrophil elastase staining and morphological features of nuclear decondensation observed by Hoechst staining. Exposure to α-defensin-1 increased neutrophil NETosis in a concentration-dependent manner, as indicated by enhanced CitH3 staining (**Figure 3A**). High-magnification images and Hoechst staining confirmed nuclear decondensation consistent with NET-associated morphology. Quantification across eight standardized fields of view showed that 10 µg/mL α-defensin-1 significantly increased the proportion of CitH3-positive cells compared with unstimulated controls (14.99 ± 1.75% vs 4.0 ± 1.28%; p < 0.002), while PMA produced a stronger response (18.51 ± 3.12% vs 4.0 ± 1.28%, p < 0.006; **Figure 3B**). In contrast, 1 µg/mL α-defensin-1 did not significantly increase NETosis (5.72 ± 1.85% vs 4.0 ± 1.28%, p = 0.6).

## Discussion

**Figure 4:**
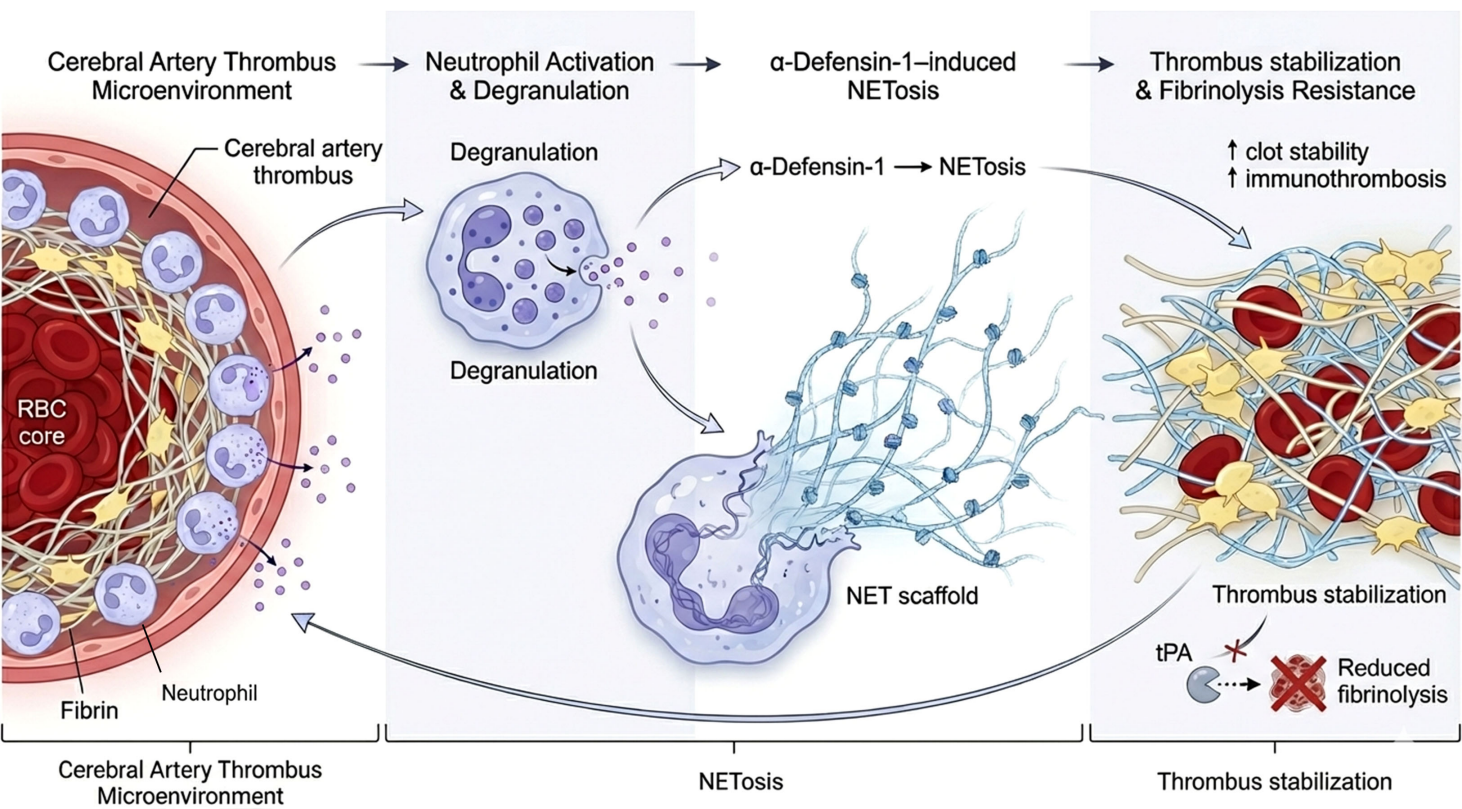
Hypothetical model of α-defensin-1-associated NET formation in stroke thrombi. Schematic illustration of a proposed role for neutrophil α-defensin-1 in stroke immunothrombosis. In the cerebral artery thrombus microenvironment, neutrophils localize mainly to leukocyte-rich regions surrounding an erythrocyte-dense core and fibrin network. Neutrophil activation leads to degranulation and release of α-defensin-1 into the extracellular space. Extracellular α-defensin-1 may promote NET formation, characterized by chromatin decondensation and extrusion of DNA-histone scaffolds. NETs interact with fibrin, platelets and red blood cells, reinforcing thrombus structure and contributing to reduced fibrinolysis. The schematic represents a graphical interpretation of the study findings and proposed immunothrombotic mechanisms. This schematic represents a hypothetical model based on the current findings and existing literature and does not reflect experimentally demonstrated mechanisms within this study.

Our findings support a potential association between neutrophil α-defensin-1 and immunothrombotic processes in ischemic stroke (**Figure 4**). We observed abundant extracellular deposition of α-defensin-1 in human stroke thrombi, predominantly in leukocyte-rich peripheral regions. This diffuse localization suggests that upon activation in the clot environment, neutrophils degranulate and release α-defensin-1, which then integrates into the thrombus matrix (13). Indeed, activation of coagulation can stimulate neutrophils to secrete α-defensins, and these cationic peptides are known to bind within tissues (for example, depositing in atherosclerotic arteries) (9, 15). Importantly, we found that this neutrophil-derived α-defensin-1 can directly induce NETosis *in vitro*, indicating a potential positive feedback loop: once a few neutrophils degranulate and release α-defensin-1, this factor can stimulate nearby neutrophils to expel NETs. NET release, in turn, may further augment thrombosis by providing structural scaffolds and activating coagulation pathways (16, 17). Consistent with this, NETs have been identified along the surfaces of stroke thrombi (especially in platelet-rich areas), where they enmesh platelets and red blood cells (18). These DNA-protein lattices not only stabilize the clot but also impede fibrinolysis; experimental studies show that while tPA can digest fibrin, platelet aggregates remain bound by the DNA scaffold of NETs unless DNase is added to dissolve it (4). In this study, although semi-quantitative image analysis demonstrated enrichment of α-defensin-1 in leukocyte-rich regions, precise quantification of its distribution at the single-cell level and its direct association with neutrophils was not possible. A further limitation is that direct co-localization of α-defensin-1 with NET markers such as CitH3 could not be performed due to antibody species incompatibility, precluding assessment of their spatial association at the single-cell level.

NETs are known to contribute to thrombus stability and resistance to lysis. In this context, our findings that α-defensin-1 is present in thrombi and can induce NET-associated changes *in vitro* suggest a potential link between neutrophil degranulation, NET formation and thrombus composition. However, the present study does not directly assess thrombus architecture, stability or fibrinolysis, and these functional implications remain to be determined.

The mechanism by which α-defensin-1 induces NETosis is not fully defined. However, several plausible pathways may be involved. As a cationic peptide, α-defensin-1 can interact with cell membranes and has been reported to modulate ion fluxes, including calcium influx, which is a known trigger of NETosis. Additionally, α-defensin-1 may promote neutrophil activation indirectly by enhancing local inflammatory signaling or by interacting with extracellular matrix components within the thrombus microenvironment. It is also possible that α-defensin-1 acts synergistically with other stimuli present in thrombi, lowering the threshold for NET formation. Further mechanistic studies will be required to delineate these pathways.

These observations extend the emerging concept of immunothrombosis in stroke (19). We add to this picture by identifying α-defensin-1 as a key neutrophil-derived factor present in all analyzed clots and capable of potentiating NETosis. This finding resonates with prior evidence of α-defensins’ pro-thrombotic activities. α-defensin levels rise dramatically during acute inflammation and have been correlated with cardiovascular events including stroke (11, 12). Functionally, α-defensins can promote platelet activation and aggregation and accelerate fibrin polymerization while impeding fibrin breakdown (13, 20, 21). Our data suggest that in stroke, α-defensin-1 released from neutrophils may serve as a bridge between these known effects: by inducing NET release, α-defensin-1 helps create a web of DNA, histones, and proteases that captures platelets and augments the fibrin network. However, as the thrombus analysis in this study is primarily descriptive, a causal relationship between α-defensin-1 and NETosis *in vivo* cannot be established from these data. Also, this study is limited by the small number of thrombi analyzed (n=6) and the clinical heterogeneity of the cohort, including differences in thrombolytic treatment, anticoagulation and comorbidities. These factors may influence thrombus composition and NET formation and therefore limit the generalizability of the findings. Although α-defensin-1 was detected in all samples, this observation should be interpreted cautiously and requires validation in larger, more homogeneous cohorts. Accordingly, these findings should not be generalized to all stroke thrombi.

We observed α-defensin-1-induced NETosis at concentrations in the microgram per milliliter range. While α-defensin levels can increase substantially during inflammation, the precise local concentrations within thrombi remain unknown; therefore, the *in vivo* relevance of these concentrations should be interpreted with caution. This autocrine/paracrine activation of neutrophils by α-defensin-1 might help explain why stroke clots are often rich in NETs and relatively resistant to lysis.

From a translational perspective, our findings highlight α-defensin-1 and NETosis as potential therapeutic targets for improving stroke outcomes. If α-defensin-1-mediated NET formation contributes to thrombus stability and thrombolytic resistance (which has not been directly addressed in this study), then disrupting this pathway may represent a targetable pathway warranting further investigation. One approach could be the neutralization or inhibition of α-defensins. These peptides are positively charged and known to be sequestered by endogenous polyanions like heparin; indeed, the presence of α-defensin-1 has been linked to heparin resistance in clot dissolution. This raises the intriguing possibility that adjunctive treatments could bind and neutralize α-defensins within clots, thereby weakening the NET-fibrin scaffold. Another strategy is to directly target NETs. Preclinical studies have already shown that combining DNase (to degrade NET DNA) with tPA can overcome the thrombolysis resistance conferred by NETs. In light of our results, inhibiting neutrophil degranulation or α-defensin-1 release might achieve a similar outcome by preventing NET formation at the outset. For example, in a mouse model, blocking neutrophil α-defensin release (via pharmacological or genetic means) resulted in smaller, more readily lysed clots, underscoring the causal role of α-defensins in thrombus propagation. Targeting α-defensin or its downstream effects could thus tilt the balance back toward thrombus resolution. It is worth noting that α-defensins have essential antimicrobial functions, so any therapeutic intervention would need to be finely timed or localized to avoid compromising host defense. Nevertheless, the concept of modulating the neutrophil-NET-thrombus axis is gaining traction, and our study identifies α-defensin-1 as a novel, tractable node in this axis.

In summary, the presence of neutrophil α-defensin-1 in human ischemic stroke thrombi and its capacity to induce NETosis provide a new insight into how innate immunity interfaces with thrombosis in the cerebral circulation. These insights not only deepen our understanding of stroke pathology but also point to new therapeutic opportunities. It should be noted, however, that these findings establish association and *in vitro* activity but do not demonstrate a causal role of α-defensin-1 in NET formation within thrombi.

In conclusion, these findings support further investigation of α-defensin-1 and NETs as potential therapeutic targets to modulate thrombus composition and improve stroke outcomes.

## Data Availability

The raw data supporting the conclusions of this article will be made available by the authors, without undue reservation.

## References

[1] Qasim R Muzammil L Qammar B Dar A Sultan L Raza M . Five-decade mortality trends in ischemic stroke in the United States: A CDC WONDER analysis. Brain Behav. (2026) 16:e71177. doi: 10.1002/brb3.71177. PMID: 41476024 PMC12755966

[2] Jolugbo P Ariens RAS . Thrombus composition and efficacy of thrombolysis and thrombectomy in acute ischemic stroke. Stroke. (2021) 52:1131–42. doi: 10.1161/strokeaha.120.032810. PMID: 33563020 PMC7610448

[3] Ducroux C Di Meglio L Loyau S Delbosc S Boisseau W Deschildre C . Thrombus neutrophil extracellular traps content impair tPA-induced thrombolysis in acute ischemic stroke. Stroke. (2018) 49:754–7. doi: 10.1161/strokeaha.117.019896. PMID: 29438080

[4] Fuchs TA Brill A Wagner DD . Neutrophil extracellular trap (NET) impact on deep vein thrombosis. Arterioscler Thromb Vasc Biol. (2012) 32:1777–83. doi: 10.1161/atvbaha.111.242859. PMID: 22652600 PMC3495595

[5] Zou S Jie H Han X Wang J . The role of neutrophil extracellular traps in sepsis and sepsis-related acute lung injury. Int Immunopharmacol. (2023) 124:110436. doi: 10.1016/j.intimp.2023.110436. PMID: 37688916

[6] Huertas-Nieto S Moraga-Yebenes A Zamora-Perez L Moreno G Maneiro-Melon N Sarnago-Cebada F . Characterization of neutrophil extracellular traps in acute myocardial infarction: A translational study. J Cardiovasc Transl Res. (2025) 18:1325–35. doi: 10.1007/s12265-025-10676-1. PMID: 40900282

[7] He W Wu Z Liu Y Ye Z . Neutrophil extracellular traps in ischemic stroke: Mechanisms, clinical implications, and therapeutic potential. Front Neurol. (2025) 16:1641985. doi: 10.3389/fneur.2025.1641985. PMID: 41018178 PMC12463598

[8] Staessens S Denorme F Francois O Desender L Dewaele T Vanacker P . Structural analysis of ischemic stroke thrombi: Histological indications for therapy resistance. Haematologica. (2020) 105:498–507. doi: 10.3324/haematol.2019.219881. PMID: 31048352 PMC7012484

[9] Nassar H Lavi E Akkawi S Bdeir K Heyman SN Raghunath PN . Alpha-defensin: Link between inflammation and atherosclerosis. Atherosclerosis. (2007) 194:452–7. doi: 10.1016/j.atherosclerosis.2006.08.046. PMID: 16989837

[10] Fu J Zong X Jin M Min J Wang F Wang Y . Mechanisms and regulation of defensins in host defense. Signal Transd Targ Ther. (2023) 8:300. doi: 10.1038/s41392-023-01553-x. PMID: 37574471 PMC10423725

[11] Joseph G Tarnow L Astrup AS Hansen TK Parving HH Flyvbjerg A . Plasma alpha-defensin is associated with cardiovascular morbidity and mortality in type 1 diabetic patients. J Clin Endocrinol Metab. (2008) 93:1470–5. doi: 10.1210/jc.2007-1910. PMID: 18211979

[12] Vordenbaumen S Sander O Bleck E Schneider M Fischer-Betz R . Cardiovascular disease and serum defensin levels in systemic lupus erythematosus. Clin Exp Rheumatol. (2012) 30:364–70. 22510487

[13] Abu-Fanne R Stepanova V Litvinov RI Abdeen S Bdeir K Higazi M. . Neutrophil alpha-defensins promote thrombosis *in vivo* by altering fibrin formation, structure, and stability. Blood. (2019) 133:481–93. doi: 10.1182/blood-2018-07-861237 PMC6356988 30442678

[14] Schneider CA Rasband WS Eliceiri KW . NIH Image to ImageJ: 25 years of image analysis. Nat Methods. (2012) 9:671–5. doi: 10.1038/nmeth.2089. PMID: 22930834 PMC5554542

[15] Ganz T . Defensins: Antimicrobial peptides of innate immunity. Nat Rev Immunol. (2003) 3:710–20. doi: 10.1038/nri1180. PMID: 12949495

[16] Rayes J Brill A . Hot under the clot: Venous thrombogenesis is an inflammatory process. Blood. (2024) 144:477–89. doi: 10.1182/blood.2023022522. PMID: 38728383

[17] Brill A Fuchs TA Savchenko AS Thomas GM Martinod K De Meyer SF . Neutrophil extracellular traps promote deep vein thrombosis in mice. J Thromb Haemost. (2012) 10:136–44. doi: 10.1111/j.1538-7836.2011.04544.x. PMID: 22044575 PMC3319651

[18] Huang G Wu H Lin B Deng D Liu Y Qu J . Targeting neutrophil extracellular traps: A new strategy for the treatment of acute ischemic stroke based on thrombolysis resistance. Semin Thromb Hemost. (2026) 52:80–91. doi: 10.1055/a-2609-3457. PMID: 40461018 PMC12799313

[19] Brinjikji W Madalina Mereuta O Dai D Kallmes DF Savastano L Liu Y . Mechanisms of fibrinolysis resistance and potential targets for thrombolysis in acute ischaemic stroke: Lessons from retrieved stroke emboli. Stroke Vasc Neurol. (2021) 6:658–67. doi: 10.1136/svn-2021-001032. PMID: 34312319 PMC8717785

[20] Horn M Bertling A Brodde MF Muller A Roth J Van Aken H . Human neutrophil alpha-defensins induce formation of fibrinogen and thrombospondin-1 amyloid-like structures and activate platelets via glycoprotein IIb/IIIa. J Thromb Haemost. (2012) 10:647–61. doi: 10.1111/j.1538-7836.2012.04640.x. PMID: 22268819

[21] Quinn KL Henriques M Tabuchi A Han B Yang H Cheng WE . Human neutrophil peptides mediate endothelial-monocyte interaction, foam cell formation, and platelet activation. Arterioscler Thromb Vasc Biol. (2011) 31:2070–9. doi: 10.1161/atvbaha.111.227116. PMID: 21817096 PMC4909471

